# Accelerating dynamic imaging of the lung using blind compressed sensing

**DOI:** 10.1186/1532-429X-16-S1-W27

**Published:** 2014-01-16

**Authors:** Sajan Goud Lingala, Yasir Mohsin, John D Newell, Jessica C Sieren, Daniel Thedens, Peter Kollasch, Mathews Jacob

**Affiliations:** 1 The University of Iowa, Iowa city, Iowa, USA; 2 Siemens Medical Solutions, Minneapolis, Minnesota, USA

## Background

Real time dynamic lung MRI is a promising tool to non-invasively assess lung function and mechanics. However, it potential is not realized in the clinic due to the restricted spatio-temporal resolution and volume coverage. The main focus of this work is to overcome these drawbacks using the recent blind compressed sensing (BCS) scheme [Lingala et al., IEEE TMI 2013], which enables recovery from undersampled k-t measurements.

## Methods

The BCS scheme exploits the sparsity of the dynamic dataset in a learned dictionary, which is estimated from undersampled measurements. Since the non-orthogonal basis functions are learnt from the data, they offer more compact representations than conventional compressed sensing (CS) schemes [Otazo et al MRM 2010] that utilize predetermined dictionaries and conventional low rank models [Lingala et al., IEEE TMI 2011] that utilize few orthogonal learned bases. We test the feasibility of the proposed scheme by recovering a retrospectively undersampled 2D free breathing lung MRI dataset using BCS. The data from a single coronal slice was acquired on an anesthetized swine using a TrueFISP sequence (TR = 138.62 msec, TE = 1.06 msec, phase encoding steps: 128, Image matrix size after interpolation: 256 × 256, FOV 320 mm2, GRAPPA factor: 3) on a 3 T Siemens Trio with a 12 channel coil. The 28 second acquisition resulted in 200 2D images with a temporal footprint of ~140 msecs/image. The reconstructed images were retrospectively undersampled using a radial k-t sampling pattern, whose spokes were uniformly distributed in a single frame. The pattern was rotated by random angles in subsequent frames to ensure incoherence. Subsampling was performed by considering 40 to 10 spokes/frame. Image reconstruction was performed by the BCS, k-t FOCUSS (CS using Fourier bases), and the low rank schemes.

## Results

The BCS scheme provided superior spatio-temporal fidelity than CS and low rank methods. The CS scheme was found to be sensitive to motion artifacts, while the low rank reconstructions suffered from spatio-temporal blurring. Feasible subsampling levels upto 25 spokes/frame where the mean square error was within 0.1 percent was achieved with the BCS scheme.

## Conclusions

The blind compressed sensing scheme utilizing learned dictionaries has potential to accelerate free breathing dynamic lung MRI. The preliminary results in this work demonstrated high fidelity reconstructions of the BCS scheme in comparison to existing compressed sensing and low rank models. Further validation on patients is required to evaluate the clinical utility.

## Funding

Grants from NSF CCF-0844812, NSF CCF-1116067, NIH 1R21HL109710-01A1, and AHA 12 PRE11920052.

**Figure 1:**
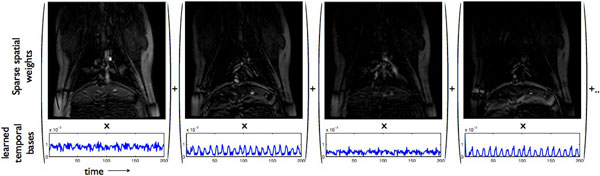
**The blind compressed sensing (BCS) model decomposition: Here, few spatial weights and its corresponding temporal basis functions are shown**. Note the weights have few non-zeros coefficients, and the learned temporal bases represent the temporal variations present in the data (eg: the second, and fourth example bases demonstrate the breathing motion, while the third demonstrate the faster cardiac pulsations.). The usage of such learned bases provide superior reconstructions compared to predetermined bases (such as Fourier bases) as demonstrated in Figure 2.

**Figure 2:**
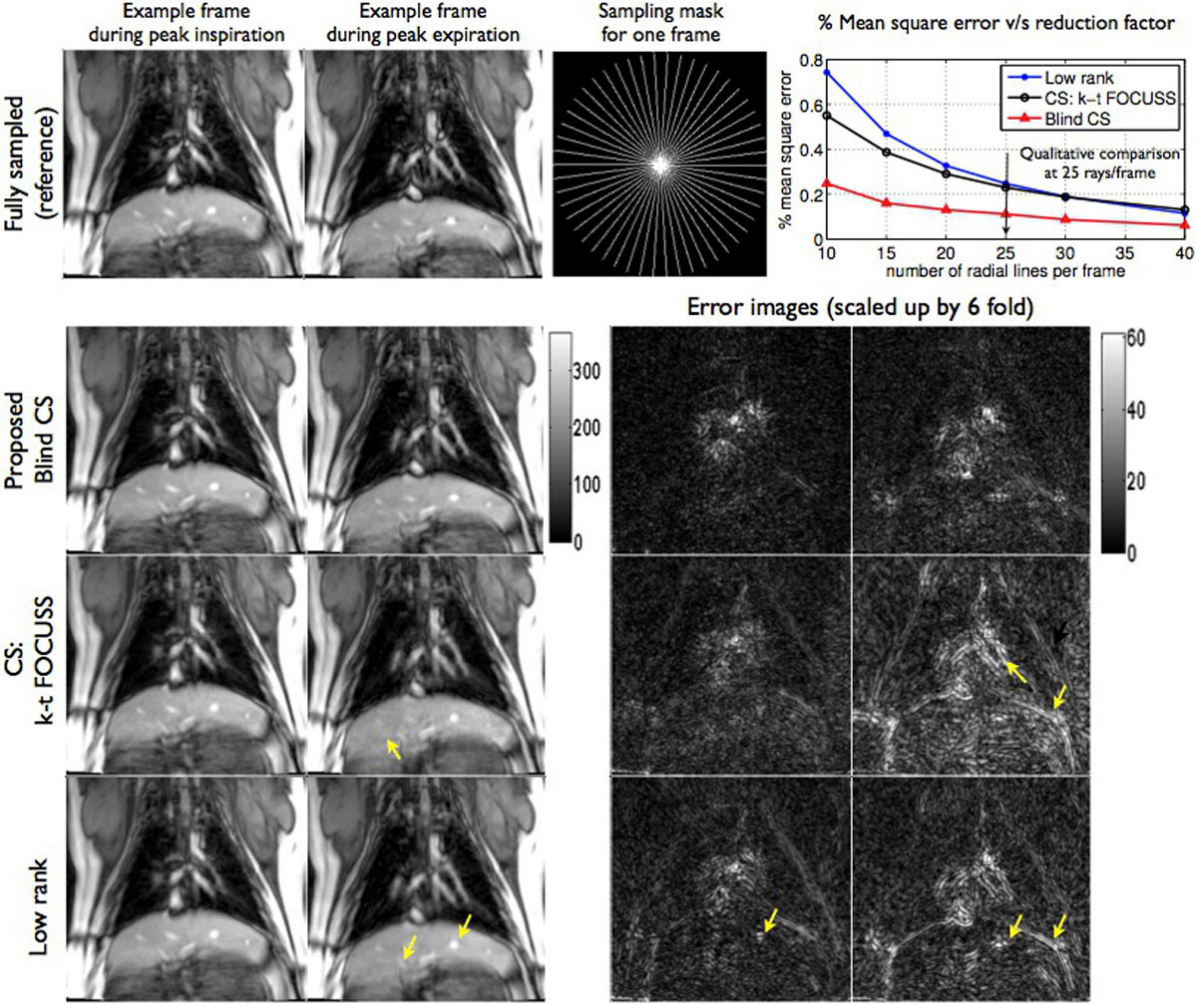
**Performance evaluation of undersampled reconstruction using the blind compressed sensing (BCS), CS and low rank schemes**: It can be seen that the proposed BCS scheme consistently produce the least mean square error at all undersampling factors. The CS, and low rank schemes depict loss in spatial features, and compromises due to motion blurring (see the yellow arrows in rows 3,4). In contrast, the proposed BCS scheme was found to be robust to motion blur, and provided superior spatio-temporal fidelity.

